# The relationship between obesity, hyperglycemia symptoms, and health-related quality of life among Hispanic and non-Hispanic white children and adolescents

**DOI:** 10.1186/1471-2296-7-3

**Published:** 2006-01-17

**Authors:** Ahmed A Arif, James E Rohrer

**Affiliations:** 1 Texas Tech University Health Sciences Center, Department of Family & Community Medicine, Division of Health Services Research, Lubbock, TX, USA; 2 Mayo Clinic Family Medicine Program/Rochester, Department of Family Medicine, 406 West Main Street, Kasson MN, USA

## Abstract

**Background:**

The current study was conducted to evaluate the effects of overweight, hyperglycemia symptoms, Hispanic ethnicity, and language barriers on health-related quality of life (HRQoL) among children and adolescents.

**Methods:**

Parents'/guardians of a population based sample of 5530 children between ages 3 and 18 were administered the parents' version of the KINDL^® ^survey instrument to assess HRQoL in children and adolescents. Multiple linear regression analysis was used to assess relationships between HRQoL, body mass index, and hyperglycemia symptoms categories.

**Results:**

The mean age of children was 10.6 (SD = 4.3). The mean KINDL^® ^total score was 79.7 (SD = 11.6) and the mean physical functioning score was 81.9 (SD = 20.3). Male children exhibited better physical health as compared to the female children (*p *< 0.001). Overweight children had lower overall HRQoL (*p *= 0.008). However, the association was not significant for the four of the six subscales including the physical health domain. Children with hyperglycemia symptoms and a family history of diabetes also had significantly lower overall and physical health HRQoL (*p *< 0.05). Children diagnosed with diabetes and in lower income strata also had significantly lower overall HRQoL (*p *< 0.05). No significant association between the Hispanic ethnicity and HRQoL was observed. However, those who reported mostly speaking Spanish exhibited significantly lower overall HRQoL (*p *= 0.001).

**Conclusion:**

Results suggest that overweight may reduce overall quality of life among children, though it does not directly influence physical functioning. However, hyperglycemia symptoms may affect both overall health and physical functioning. Findings also suggest the need for developing programs directed at overcoming language barriers that may face Spanish-speaking children or their parents. Furthermore, targeting children who have hyperglycemia symptoms with public information campaigns may be more appropriate than targeting overweight children.

## Background

The prevalence of both obesity and diabetes has been increasing among children in the U.S. over the last several years [1,2]. Hispanic children have especially been affected by this problem [2,3]. This trend is expected to continue indefinitely unless effective public health strategies can be implemented to combat the epidemic. Health-related quality of life (HRQoL), which describes the effect of diseases and illnesses on persons' physical, social, and mental well being, is widely recognized as an important health outcome which can be used to evaluate medical care treatments and monitor community health [4,5]. However, little research has focused on HRQoL among children, partly because of limited number of available validated instruments that measures HRQoL among children and adolescents. The precise relationship between obesity, diabetes, and HRQoL among children has been difficult to elucidate partly because pediatric diabetes is assumed to be under-diagnosed and self-reports of diabetes prevalence are usually an underestimates. Even if hyperglycemia symptoms are used as proxy measures for diabetes, other unanswered questions needs to be addressed. Is overweight directly related to pediatric HRQoL when hyperglycemia symptoms are controlled? Is Hispanic ethnicity directly related to pediatric HRQoL when language preference is controlled? And, is Hispanic ethnicity directly related to pediatric HRQoL when psychosocial aspects of the child's health are taken into account? The study reported addressed these questions using KINDL^® ^HRQoL survey instrument.

## Methods

### Study sample

The childhood health and diabetes survey is a population based telephone survey of 5933 households residing in 109 counties that comprise west Texas [6]. The survey was conducted in 2002. A survey research firm conducted a pretest with 40 qualified respondents (having children over age 3 and under age 19) in West Texas. The pretest was conducted in both English and Spanish languages. The instrument was translated into Spanish by one translator. Upon completion, experienced Spanish-speaking interviewers provided feedback for possible revisions until a consensus was achieved on the translation. Pretest interviews were monitored for observations relative to respondent interest, comprehension and other factors that could impact the validity and reliability of measurements and changes were made to the instrument as necessary before the full-scale interviewing effort began. The sample for the Survey of Childhood Diabetes was a telephone list-assisted random digit dialing (RDD) sample of the area codes and telephone exchanges used in 111 Texas counties defined as the West Texas Region. An RDD sample of this type is generated by first identifying each area code and telephone exchange used in the targeted geographic region in direct proportion to number of listed residential households. Using listed residential households as a guide, the last four numbers of the telephone number were randomly generated for telephone banks where a "100 block" (the last three digits of a phone number) has at least two listed residential phone numbers. All residents age 18 years or older who have telephones comprised the sampling frame. Unlisted telephone numbers were included in the sample. The sample was purchased from Genesys Sampling Systems, a nationally recognized sampling firm. Each phone number in a sample was called until a final disposition designating that numbers should no longer be called was assigned or until a number had been tried at least five times. The response rate was 54.7% among households known to have age-eligible children. The cooperation rate was 70.4 percent. Cooperation rate was calculated as the percentage of households identified through the screening process to be part of the target population that completed the survey compared to percentage of these households that refused to participate. Of the 5933 respondents, 5530 (93.2%) were either Hispanics (43.2%) or non-Hispanic whites (50.0%) and were included in this analysis.

### Dependent variable

The parents' version of the pediatric quality of life (QoL) scale, KINDL^® ^survey instrument was used to assess HRQoL in children and adolescents. This survey instrument is available for use with permission from developers [7]. This 24 item generic instrument has six sub-scales: physical functioning, emotional well-being, self-esteem, family, friends, and school. The response scale is from 1 (never) to 5 (all the time) and is based on a four week recall. The summary scores of the total and the six subscale KINDL^® ^subscales were computed and transformed (range: 0 lowest to 100 highest) using the algorithm provided by the developer. Higher scores indicate better health. The reliability and validity of the KINDL^® ^questionnaire was previously estimated among children with and without chronic illnesses, including diabetes, and was found to be satisfactory [8,9].

### Measures

During the survey, parents'/guardians of children between 3 and 18 years old were interviewed by a bilingual interviewer about theses measures: child's height and weight, which were used to compute body mass index (weight in kg/height in meters^2^) in the form of percentiles (based on the BMI-for-age growth charts) according to the Centers for Disease Control (CDC) guidelines [10]: normal = ≥5^th^–<85^th ^percentile (pct); underweight = <5^th ^pct, at-risk for overweight = 85^th^–95^th ^pct, and overweight = >95^th ^pct; hyperglycemia symptoms-: responding parents were asked if their child had any of the following symptoms [11]: frequent urination (polyuria), always hungry (polyphagia), always thirsty (polydipsia), weight loss in spite of increased appetite, blurred vision, and increasing fatigue. These symptoms were summed and categorized into none, one-two, three-four, and five or more symptoms; parents'race/ethnicity (Hispanics, non-Hispanic white); annual family income (<20,000, 20–40 k, 40–60 k, and 60 k+); child ever diagnosed by a health care provider to have diabetes (yes/no); family history of diabetes; and parents'/guardians acculturation status (mostly speak English, mostly speak Spanish, Both, and Other languages).

### Statistical analysis

Mean KINDL^® ^total scores and the six sub-scale scores were computed for each BMI and hyperglycemia categories. In addition, mean KINDL^® ^scores were also computed for those who reported that their children were diagnosed with diabetes. Median KINDL scores for the overall and six subscales were also computed. Multiple linear regression analysis was used to assess relationships between the KINDL^® ^total score, the six subscales, BMI categories, and hyperglycemia symptoms. Cronbach alpha was used to determine internal consistency for the overall and subscale scores. All analyses were performed using STATA version 9.0 statistical software package (College Station, TX, 2005). The study was approved by the institutional review board.

## Results

The mean age of children was 10.6 years (SD = 4.3); 51% were male; and 20% were considered as overweight (BMI>95^th ^percentile). A total of 41 children (0.74%) were diagnosed with diabetes. Approximately 13% of children reported three or more symptoms of hyperglycemia and more than 50% had a family history of diabetes.

The mean (SD) KINDL^® ^total score was 79.7 (11.6) and the mean (SD) scores for the six subscales range from as low as 74.7 (19.5) for self-esteem to as high as 85.9 (15.1) for the emotional well-being (Table 1). The mean KINDL^® ^scores were generally higher for the physical functioning and psychological well-being sub-scales. The mean scores did not vary for the various BMI categories; however, a dose response relationship was observed for hyperglycemia symptoms with a gradual decline in the mean KINDL^® ^scores with increasing number of symptoms reported. Similarly, those who were diagnosed with diabetes had lower mean KINDL^® ^scores as compared to those without the diagnosis of diabetes (Table 1). The overall internal consistency (as measured by Cronbach alpha) was 0.74. It ranged from as low as 0.41 for school subscale to as high as 0.76 for physical functioning subscale. The scores were very similar among males and females (Table 2).

**Table 1:** Mean (SD) KINDL^® ^scores among Hispanic and non-Hispanic white children by BMI categories, hyperglycemia symptoms, and diabetes status

	**KINDL Total Score**	**Physical functioning**	**Emotional well-being**	**Self-esteem**	**Family**	**Friends**	**School**
	*Mean (SD)*	*Mean (SD)*	*Mean (SD)*	*Mean (SD)*	*Mean (SD)*	*Mean (SD)*	*Mean (SD)*

**All^a^**	79.7 (11.6)	81.9 (20.3)	85.9 (15.1)	74.7 (19.5)	76.9 (17.1)	80.8 (15.2)	77.3 (17.4)
**Median scores**	81.3	87.5	87.5	75.0	75.0	81.3	81.3
**BMI categories^b^**							
Normal (n = 2,228; n = 2096^c^)	79.5 (11.6	81.1 (21.0)	86.1 (14.4)	75.2 (18.8)	76.2 (16.9)	81.4 (14.5)	77.0 (16.8)
Underweight (n = 437; n = 385^c^)	80.2 (11.9	83.7 (19.2)	86.7 (15.3)	75.6 (19.4)	75.0 (16.9)	80.6 (15.2)	79.8 (16.7)
At-risk of overweight (n = 634; n = 586^c^)	78.9 (11.7	81.7 (19.5)	85.7 (15.0)	74.3 (18.9)	75.3 (17.1)	79.3 (15.0)	75.9 (18.1)
Overweight (n = 1099; n = 958^c^)	79.2 (11.9	80.7 (21.0)	85.8 (15.6)	74.5 (20.0)	75.7 (17.3)	80.1 (15.7)	78.0 (18.2)
							
**Hyperglycemia symptoms^d^**							
None (n = 1797; n = 1633^c^)	83.4 (9.7)	88.0 (16.2)	89.7 (12.5)	78.4 (18.5)	80.6 (15.5)	82.9 (13.7)	80.4 (16.1)
1–2 (n = 2746; n = 2491^c^)	79.4 (11.1)	81.2 (20.3)	85.8 (14.5)	74.6 (18.9)	76.2 (17.2)	81.0 (14.8)	77.3 (17.2)
3–4 (n = 667; n = 621^c^)	72.8 (12.6)	71.0 (22.9)	78.9 (17.8)	68.2 (21.2)	71.2 (17.8)	76.2 (17.4)	71.5 (18.3)
5 or more (n = 263 n = 28^c^)	63.7 (14.2)	57.4 (20.4)	69.6 (19.9)	56.9 (18.9)	68.8 (17.8)	69.4 (16.9)	59.1 (19.6)
							
**Diagnosed with Diabetes**							
No (n = 5476; n = 4980^b^)	79.7 (11.5)	82.0 (20.3)	86.0 (14.9)	74.8 (19.5)	77.0 (17.1)	80.9 (15.1)	77.4 (17.4)
Yes (n = 41; n = 40^b^)	72.2 (16.8)	71.2 (23.7)	75.6 (25.3)	66.3 (24.4)	75.3 (19.5)	75.1 (24.4)	69.7 (18.0)

SD = Standard deviation

^a ^n varies: KINDL total score n = 5515; physical functioning n = 5521; emotional well-being n = 5513; self-esteem n = 5501; family n = 5514; friends n = 5491; and school n = 4989.

^b ^BMI Categories: Normal = ≥5^th^–<85^th ^percentile (pct); Underweight = <5^th ^pct; At-risk for overweight = 85^th^–95^th ^pct; Overweight = >95^th ^pct.

^c ^n include only those children who go to school

^d ^41 children who had diabetes were excluded from this group

**Table 2:** Internal consistency of KINDL^® ^HRQoL questionnaire items

	**No. Items**	**Cronbach alpha**		
		**All**	**Male**	**Female**

Total Score	24	0.73	0.73	0.74
Physical well-being	4	0.76	0.75	0.76
Emotional well-being	4	0.52	0.53	0.52
Self-esteem	4	0.74	0.74	0.73
Family	4	0.59	0.60	0.59
Social contacts	4	0.56	0.58	0.55
School	4	0.41	0.38	0.44

Tables 3 and 4 present results of the multiple linear regression analyses of the KINDL^® ^total score and the six subscale scores. Older children had lower KINDL^® ^total scores. Male children exhibited better health on the physical functioning subscale as compared to the female children (*p *< 0.001). The lower KINDL^® ^total score among overweight children indicated impaired QoL (*p *= 0.008). However, the association was not significant in the four of the six subscales including the physical functioning domain. Overweight was associated with lower self-esteem and lower score on friends subscale (Table 4). A negative association between the KINDL^® ^total score and hyperglycemia symptoms was observed, with children reporting more hyperglycemia symptoms less likely to be perceived by their parents' as healthy as reflected by the decreasing KINDL^® ^total scores (*p *< 0.001). A similar trend was observed for all the six subscales. Children with diabetes and those with a family history of diabetes also had a lower perceived quality of life. Similarly, those in the lowest income strata and those who spoke mostly Spanish had lower KINDL^® ^total scores. In the physical functioning and family subscales, those who reported mostly speaking Spanish were more likely to report that there children were healthy (*p *< 0.001). However, they were more likely to rate their child's health as poor on emotional well-being (*p *< 0.001), self-esteem (*p *< 0.001), friends (*p *< 0.05), and school (*p *< 0.001) subscales.

**Table 3:** Multiple linear regression analyses of parent reports of overall HRQoL

	**KINDL^® ^Total Score**		
	
	**β**	**95% Confidence Interval**	***p*-value**
**Age of child **(years)^a^	-2.46	-2.83, -2.10	<0.001
**Sex**			
(Female)			
Male	0.15	-0.44, 0.74	0.621
**Body Mass Index**			
(Normal)			
Underweight	-0.63	-1.76, 0.50	0.272
At-risk of overweight	-0.66	-1.64, 0.31	0.183
Over weight	-1.15	-2.00, -0.30	0.008
**Hyperglycemia symptoms**			
(None)			
1–2 symptoms	-3.88	-4.53, -3.23	<0.001
3–4 symptoms	-10.13	-11.10, -9.16	<0.001
5 or more symptoms	-19.24	-23.06, -15.42	<0.001
**Race/Ethnicity^b^**			
(Non-Hispanic White)			
Hispanic	1.46	0.65, 2.27	<0.001
**Family history of diabetes**			
(No)	-0.74	-1.34, -0.15	0.014
**Child diagnosed with diabetes**			
(No)	-3.81	-7.24, -0.38	0.03
**Annual income**			
(60 k+)			
<20 k	-1.74	-2.71, -0.77	<0.001
20–40	-0.59	-1.47, 0.28	0.184
40–60	-0.74	-1.69, 0.22	0.132
**Acculturation**			
(Mostly speak English)			
Mostly speak Spanish	-1.88	-2.98, -0.78	0.001
Speak both languages equally	0.10	-0.93, 1.12	0.853
Speak other language	-2.64	-8.71, 3.42	0.393

Note: Variables in parentheses are referent categories. The model is adjusted simultaneously for all variables included in the model.

^a ^Age of the child is reported in increments of 5 years.

^b ^Responding parents' race/ethnicity

**Table 4:** Multiple linear regression analyses of parent reports of the six KINDL^® ^sub-scale scores

	**Physical functioning**	**Emotional well-being**	**Self-esteem**	**Family**	**Friends**	**School**
**Age of child **(years)^a^	-1.80***	-2.12***	-3.46***	-0.68*	-0.76**	-6.23***
**Sex**						
(Female)						
Male	2.56***	0.61	-0.76	-0.52	0.27	-1.27**
**Body Mass Index**						
(Normal)						
Underweight	1.41	-0.54	-1.19	-1.99*	-1.45	0.47
At-risk of overweight	1.02	-0.17	-0.86	-1.10	-2.00**	-1.11
Over weight	-0.87	-0.54	-2.22***	-0.71	-1.39*	-0.94
**Hyperglycemia symptoms**						
(None)						
1–2 symptoms	-6.77***	-3.93***	-3.74***	-3.99***	-1.84***	-3.02***
3–4 symptoms	-16.85***	-10.40***	-9.65***	-9.11***	-6.62***	-7.85***
5 or more symptoms	-30.64***	-21.79***	-20.62***	-11.85***	-13.02***	-18.53***
**Race/Ethnicity^b^**						
(Non-Hispanic White)						
Hispanic	1.14	0.58	2.93***	2.35***	1.83***	-0.56
**Family history of diabetes**						
(No)	-2.31***	-0.77	0.37	-0.55	-0.34	-0.66
**Child diagnosed with diabetes**						
(No)	-5.52*	-5.65*	-4.92	0.81	-4.00	-4.10
**Family income**						
(60 k+)						
<20 k	-0.65	-1.56*	-1.91*	-2.31**	-1.61*	-2.88***
20–40	-0.42	0.37	-0.71	-2.11**	-0.42	-1.14
40–60	-0.16	-0.32	-1.77*	-1.06	-0.67	-0.83
**Acculturation**						
(Mostly speak English)						
Mostly speak Spanish	2.38*	-4.72***	-8.55***	8.63***	-1.72*	-7.41***
Speak both languages equally	1.79	-1.42*	-1.00	2.93***	0.43	-1.97*
Speak other language	-1.99	-5.11	-11.77*	5.54	0.59	0.04

Note: Variables in parentheses are referent categories. Each model is adjusted simultaneously for all variables. The model for the school sub-scale include only those children who go to school

^a ^Age of the child is reported in increments of 5 years.

^b ^Responding parents' race/ethnicity

* p = 0.05, ** p = 0.01, ***p = 0.001

## Discussion

The results of this study challenge the possible assumed relationship between Hispanic ethnicity and health-related quality of life among children. In our study, both overall HRQoL and physical health were inversely related to having been diagnosed with diabetes, having a family history of diabetes, and having hyperglycemia symptoms. However, while being overweight was inversely related to overall HRQoL it did not have an independent significant relationship with physical functioning in this cross-sectional sample. There is a paucity of literature addressing QoL issues among overweight children and adolescents. Ravens-Siebere et al. [12] used the KINDL^® ^questionnaire to assess QoL among obese German children. An impaired QoL on all subscales, with the exception of the physical functioning was observed, similar to the results reported in this study. Hesketh et al. [13], using data from Child Health Questionnaire (CHQ), reported that increased frequency of diabetes symptoms was associated with poor QoL on physical and psychosocial functioning scales. On the other hand, Wagner and colleagues [14] failed to observe any relationship between body mass index and any of the scales measuring KINDL^® ^HRQoL among children with type 1 diabetes mellitus. Wagner et al's study population had access to a supportive network and an experienced multidisciplinary team of health care professionals managing their diabetes, which may explain the absence of any association with psychosocial outcomes. Our results suggest that overweight does not directly influence physical functioning but may have an indirect effect via self-esteem domain and/or hyperglycemia symptoms. Efforts should be made for early psychosocial intervention and early detection and control of hyperglycemia via timely primary care for maintaining better physical health among children.

Speaking Spanish was significantly related to HRQoL in complex ways. Those who mostly spoke Spanish at home rated their children health poorer on overall, emotional, self-esteem, friends, and school subscales as compared to those who reported speaking mostly English (*p *< 0.001). However, they rated their children health better on physical health and family subscales. Interestingly, Hispanic ethnicity was not significantly related to physical health, emotional well-being, or school subscales when language ability was controlled. This suggests that Hispanic ethnicity does not directly determine poor physical health among children. However, because of small numbers the role of chance cannot be ruled out. Hence, overall KINDL^® ^score may be more relevant for this finding. The results of our study suggest that efforts should be made to overcome language barriers that may face Spanish-speaking children or their parents.

Although we cannot infer causality because of the cross-sectional design of our study, it seems likely that hyperglycemia symptoms lead to poor HRQoL. The West Texas population is not typical of the U.S. population and thus results may not be generalizable. However, our findings may be relevant to other regions where the region is largely rural, many areas are medically underserved, and many children are not as conversant in English as they are in Spanish. The low response rate of approximately 55% can potentially result in self-selection bias if parents concerned with their child's health were more likely to participate in the survey. However, the rate of participation was comparable to other population-based health surveys [15]. Similarly, since this was a telephone survey, exclusion of subjects without telephone may induce a potential bias in the study. This seems unlikely though since less than 5% of households in Texas are without telephone service [16]. In this study parents'/guardians acted as proxy for children and may not have reported their child's health accurately. It is difficult to determine the direction of bias this may have caused in this cross-sectional study.

The impact of hyperglycemic symptoms and language barriers on the health of children in the United States is potentially quite large. The increasing prevalence of childhood obesity has been recognized, but this fact understates the true magnitude of the problem. At least among rural and Spanish-speaking children, hyperglycemia symptoms are much more common and have more direct impact on health.

## Conclusion

In conclusion, our results show that children with obesity and hyperglycemia have poor HRQoL. Programs should be directed at children who have hyperglycemia symptoms. Progams also should be designed to overcome language barriers facing Spanish-speaking children or their parents. The results of this study further our understanding of complex inter-relationship between obesity, hyperglycemia symptoms, Hispanic ethnicity, and language barriers and their effect on HRQoL which could help guide the effective allocation of resources and targeting of public health initiatives.

## Competing interests

The author(s) declare that they have no competing interests

## Authors' contributions

AA carried out the study as part of funding from the Centers for Disease Control and Prevention, performed statistical analyses and drafted the manuscript. JER participated in the design of the study, draft of the manuscript, and interpretation of results.

## Pre-publication history

The pre-publication history for this paper can be accessed here:


